# Children with cancer at the end of life in a middle-income country: integrated pediatric palliative care improves outcomes

**DOI:** 10.1186/s12904-024-01354-1

**Published:** 2024-02-02

**Authors:** María Isabel Cuervo-Suarez, Daniela Cleves, Natalia Duque-Nieto, Angélica Claros-Hulbert, Karen Molina-Gómez, Jhon Edwar Bolaños-Lopez, María Elena Tello-Cajiao, Justin N Baker, Michael J. McNeil, Ximena García-Quintero

**Affiliations:** 1 Palliative Care Department, Fundación Valle del Lili, Avenida Simón Bolívar. Cra. 98 No.18–49, Cali, 760032 Colombia; 2 Facultad de Ciencias de la Salud, Universidad Icesi, Cali, 760031 Colombia; 3 Department of Global Pediatric Medicine, St. Jude Children’s Hospital, Memphis, TN 38105 USA; 4 Department of Pain and Palliative Care, Grupo Keralty, Clinica Sebastian de Belalcazar, Cali, 760044 Colombia; 5 Faculty of Health Sciences, Universidad de la Sabana, Chia, 250001 Colombia; 6 Centro de Biociencias, Seguros SURA Colombia, Medellín, 050021 Colombia; 7 Division Chief, Quality of Life and Pediatric Palliative, Stanford University School of Medicine, Alto, CA 94304 USA

**Keywords:** Palliative Care, Cancer Care Units, Pediatrics, End-Of-Life Care

## Abstract

**Background:**

In 2020, the Global Cancer Observatory reported 280,000 cases of childhood cancer worldwide, with a higher burden of disease and mortality rates in low- and middle-income countries. In 2022, the National Institute of Health reported 1708 new cases of childhood cancer in Colombia and an overall survival rate of approximately 55%. The aim of this study is to compare outcomes in children with cancer in the hospital setting during the last 72 h of life who received concurrent Pediatric Palliative Care (PPC) versus oncology care alone.

**Methods:**

An observational descriptive study was conducted between January 2013 and June 2022 in a center for pediatric patients with oncological diagnoses. In 2017, the PPC team was created. Patients between 28 days and 17 years of age who were hospitalized at least 72 h before death were included. A retrospective review of the medical records of patients in the last 72 h of life was performed. Two cohorts were established: oncology-alone group received exclusive management by oncology, and oncology and PPC received concurrent oncology and PPC management since the diagnosis.

**Results:**

We evaluated 257 medical records of deceased pediatric patients with cancer diagnoses. For the first cohort (2013–2017), 136 patients were included; for the second cohort (2018 and 2022), 121 patients were evaluated. The most frequent diagnosis was leukemia [47.1% (*n* = 121)]. No significant difference was found in either group between dyspnea, pain, and seizures. Dyspnea was the most frequent symptom in both groups. Agitation and anxiety were reported more frequently in children from the oncology-alone group (22.1% and 13.2%, respectively). The oncology and PPC group received more psychology and social work consultation (94.2% and 70.2% vs. 84.6 and 54.4% in the oncology alone group) and had a higher percentage of advance care planning (79.3% vs. 62.5% in the oncology alone group).

**Conclusions:**

This retrospective study highlights that PPC at the end of life (EoL) offers a holistic approach to the physical and psychosocial symptoms experienced by children with cancer; these patients received more comfort through symptom management and less aggressive treatment at the EoL. The availability of a PPC team may contribute to improvements in the quality of end-of-life care.

**Trial registration:**

retrospectively registered.

**Supplementary Information:**

The online version contains supplementary material available at 10.1186/s12904-024-01354-1.

## Introduction

The incidence of pediatric cancer remains very high around the globe. In 2020, the Global Cancer Observatory reported 280,000 cases of childhood cancer worldwide, with a higher burden of disease and mortality rates in low- and middle-income countries (LMICs) [1–5]. In Colombia, a middle-income country (MIC) in Latin America (LA), the Childhood Cancer Outcomes Surveillance System (VIGICANCER) and the International Agency for Research on Cancer (GLOBOCAN) reported that the pediatric cancer incidence rate between 2015 and 2020 was 14.2 cases per 100,000 inhabitants [6].

In 2022, the National Institute of Health reported 1708 new cases of childhood cancer in Colombia and an overall survival rate of approximately 55% [7]. These rates are increasing over time, and it is important to ensure that these patients receive a multidisciplinary and holistic approach, considering not only disease-directed therapy but also their global well-being throughout the trajectory of their illness from diagnosis [8, 9].

PPC is defined as the prevention and relief of physical, psychosocial, and spiritual suffering of pediatric patients and their families facing a life-threatening disease [10, 11]. Evidence suggests that early implementation of PPC improves quality of life (QoL) for children with cancer and their families, reduces symptom burden, diminishes costs of care at EoL, and decreases the need for intensive care at the EoL, with more patients dying outside of the hospital [12, 13]. Approximately 4 million children worldwide require PPC [14, 15].

Although there is knowledge regarding both the need and, to a large extent, how care should be provided, many professionals receive no training in PPC, and many feel uncomfortable addressing patient symptoms and/or grief and bereavement for the patient’s family [5, 16]. Children in MICs are less likely to have access to PPC due to a lack of awareness among pediatric oncologists about PPC and the limited availability of Palliative Care (PC) services [17].

A systematic review by Cheng et al. in the United States underscores that integration of PC in oncology patients often encounters delays, primarily due to late discussion in the illness trajectory. This gap between the initial conversation on PC and its actual implementation poses a significant challenge [18]. Additionally, they describe that only 54% of oncology patients received any form of palliative care prior to death, indicating substantial barriers in accessing PPC [18]. A separate systematic review conducted in Europe focused on the early integration of PPC in oncologic patients suggests that children with and without access to PPC may experience a lower QoL during EoL situations [19]. However, those who received an intervention one month prior to death had a greater positive impact on QoL [19].

Regarding Latin America, a recent study comparing the ideal timing of PPC integration with the actual implementation for children with cancer provides a thorough assessment of several various barriers [20]. These encompass limited capacity for home-based services, limited access to PPC support, limited physician knowledge, physician discomfort in discussing PPC, and concerns about familial resistance to PPC involvement [20], Displaying the need to identify and reduce disparities in PPC for children with cancer in Latin America. Currently, the Colombian Observatory in Palliative Care determined that 26% of the overall oncological population in Colombia necessitates PC during the EoL phase. This classification designates region exhibiting a markedly heightened prevalence of potential PC requirements. Specifically, pediatric deaths constituted 1.2% of this demographic [21].

We aim to compare the outcomes surrounding the deaths of hospitalized pediatric cancer patients who received specialized PPC versus those who received nonspecialized care at a high-complexity center in a tertiary hospital located in a MIC, specifically Colombia.

## Methods

### Design and patient selection

**Fig. 1:**
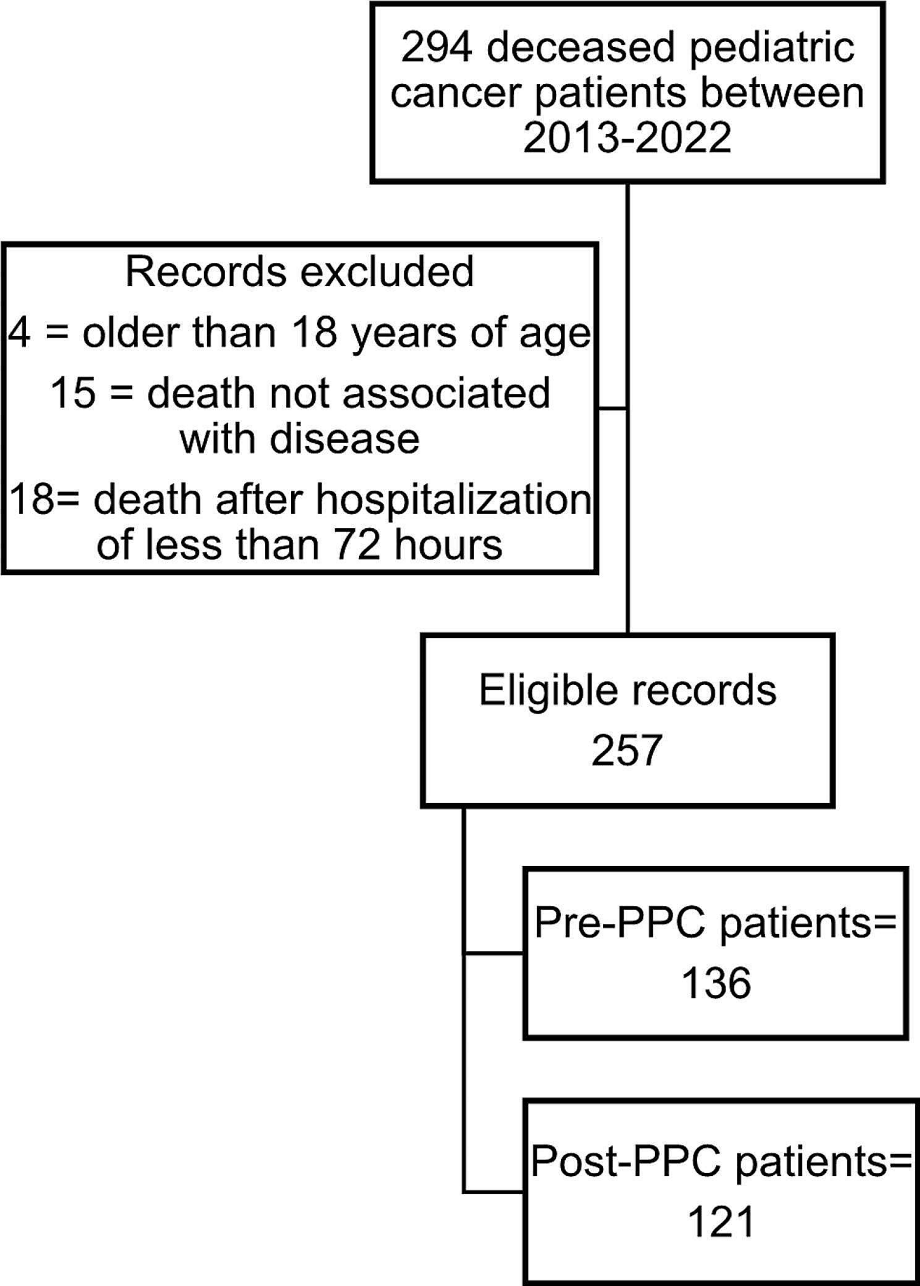
Flow chart of patient selection

An observational descriptive study was conducted between January 2013 and June 2022 at the Hospital Universitario Fundación Valle de Lili (FVL), located in the city of Cali, Colombia. The FVL is a high-complexity referral center for pediatric patients with oncologic diagnoses in southwestern Colombia. At the beginning of the study, the hospital had a total of 510 beds, of which 177 were for pediatric patients, including 30 in the pediatric intensive care unit (PICU) and 41 in the neonatal intensive care unit (NICU). By the end of the study, the hospital had a total of 668 beds, of which 230 were for pediatric patients, including 40 in the PICU and 41 in the NICU. Patient selection is presented in Fig. 1. The study included all patients between 28 days of age and 17 years of age with an oncologic diagnosis (hematologic or solid tumors) who died at FVL. The exclusion criteria were patients older than 18 years of age, deaths not associated with disease (e.g., trauma, other etiologies not associated with oncologic disease or treatment), and death after hospitalization of less than 72 h. Data collection was based on the retrospective review of medical records by the palliative care research team. The information was recorded in the institutional digital database (BDClinic©).

### The institutional pediatric palliative care program

In 2017, the Pediatric Palliative Care Program called “Taking Care of You” was created at FVL. It included a multidisciplinary team of clinicians: pediatrician, family doctor, nurse, psychologist, social worker, and spiritual advisor, all with specialized training in PPC. The year 2017 was a year of implementing the program by advocating about the benefits of palliative care in the different pediatric services. The program intended to provide comprehensive and continuous PC through a multidisciplinary intervention to meet the needs of children and families with complex and life-limiting chronic diseases and aimed to improve patient and family QoL. The approach to the patient was carried out by implementing an integrative model, which consists of all the members of the palliative care team working together with professionals in psychology, social work, and spiritual chaplain, approaching biopsychosocial needs such as family resources, family problems, social support, patient and family preferences and supporting decision-making. The program provides outpatient and inpatient services; home care is covered by other healthcare providers outside the institution. The palliative care service is available to all patients and pediatricians of the institution by telephone 24 h a day, 7 days a week, and in person during the day from Monday to Sunday 8 h a day. The care of hospitalized patients during the evening hours is provided by general pediatricians with the support of the palliative care physician by telephone.

After the PPC program was implemented, in alignment with national and institutional policies and previous agreements with the pediatric oncology team, all patients were concurrently followed up by oncology and PPC from diagnosis. This early integration of PPC in children with cancer started with an initial multidisciplinary assessment. The PPC team participated weekly in the pediatric oncology meeting, and according to the needs presented at the meeting by an interdisciplinary team (oncology, nursing, psychology, social work, rehabilitation therapy), the intensity of follow-up by PPC was defined and was determined by several factors, such as the diagnosis, prognosis, presence of symptoms, and psychosocial needs found at the beginning of the evaluation.

### Oncology alone and oncology and PPC cohorts

Two temporal cohorts of patients were identified. The first was composed of those patients who received exclusive care from the oncology team between January 1, 2013, and December 31, 2017. Patient care was performed by the oncology team. The second cohort was defined as patients who received concurrent oncology and PPC management between January 1, 2018, and June 30, 2022, when the institutional PPC team was already established. This group of patients was followed by both oncology specialists and the PPC team from diagnosis to EoL (Oncology and PPC group).

### Variables and data collection

Data collection included sociodemographic information, clinical characteristics, and medical and psychosocial interventions at EoL. The EoL period was defined as the 72 h before death. Description of the support and EoL conditions of the selected patients was the main outcome assessed. Variables related to the disease were type of tumor (solid, leukemia, lymphoma, nervous system tumor or rare childhood neoplasm) and the objective of the oncologic treatment (curative or palliative intention). Continuity of chemotherapy in the patient was not assessed.

The selection of symptoms for the study was based on those most commonly reported in the literature, including pain, dyspnea, convulsions, agitation, prolonged crying, anxiety, and fear [22]. These variables were measured as present or absent. Other variables included place of death (inpatient, pediatric intensive care unit, or emergency department), life-sustaining treatment and limited life support. (Appendix 1)

The EoL period was defined as the 72 h before death. A retrospective review of the medical records of the last 72 h of life (definition of EoL) of the patients was performed by the multidisciplinary team to identify patient symptoms and interventions. Life-sustaining treatment was defined as patients who needed cardiopulmonary resuscitation (CPR), cardioversion, intubation, or code doses of medications and life-sustaining technology, while limited life support included both the withdrawal or withholding of life-sustaining measures and do-not-resuscitate (DNR) orders. Withdrawal of treatment was defined as discontinuation of the life-sustaining intervention/treatment that was already in place, while withholding treatment was defined as the ‘non-initiation’ or the decision not to escalate a life-sustaining treatment. The data were collected by six physicians who were part of the research team of the study, collecting all the information from the patient’s medical records for the last 72 h prior to death. The research protocol was reviewed and approved by the Institutional Ethics Committee of the FVL (Protocol No. 1160).

### Statistical analysis

Comparisons between the oncology alone and oncology and PPC groups were made using the variables collected. Categorical variables were presented in tables of absolute and relative frequencies, comparing them using the z test, Chi2 test or Fisher exact test. Integer variables were summarized with medians and interquartile ranges (IQR) and compared by the Mann‒Whitney U test. All estimates were made using 95% Confidence Intervals (95% CI); *p* values < 0.05 were considered statistically significant. Calculations were performed with STATA 15 (StataCorp, College Station, TX, USA).

## Results

We evaluated the medical records of 294 pediatric patients who died of cancer in the FVL between 2013 and June 2022. Two cohorts with 257 subjects were established. The 2013 to 2017 cohort included 136 patients and constituted the Oncology Alone group. The cohort from 2018 to 2022 consists of 121 patients corresponding to the Oncology and PPC group. Thirty-seven patients were excluded for reasons such as deaths not associated with disease (e.g., trauma, other etiologies not associated with oncologic disease or treatment) and death after hospitalization of less than 72 h.

### Patients and interventions

Patient characteristics by intervention group are shown in Table 1. In both groups, the median age was 10 years (IQR 4–14 years old), and 58.75% were male (*n* = 151). The most common diagnosis was leukemia (46.30%, *n* = 119); most patients received treatment with curative intent (52.7%, *n* = 150).

Dyspnea was the most frequently reported symptom in both groups (54.08%, *n* = 139), followed by pain (47.85%, *n* = 123), with no statistical differences between them. In contrast, agitation and anxiety were reported less frequently in the oncology and PPC group compared to those who received oncology care alone (22.1% *n* = 30 and 13.2% *n* = 18, *p: 0.01 and p: 0.05* respectively).

Regarding interventions, opioids were used to manage symptoms in 89% of treated patients (*n* = 230). The cohort of concurrent management by Oncology and PPC group received more psychology (94.2%, *n* = 114, *p* = 0.01) and social work (70.2%, *n* = 85, *p* = 0.006) interventions. In addition, there was a higher percentage of advance care planning in patients undergoing concurrent care compared to the patients attending by the Oncology alone Group (79.3% *n* = 96 vs. 62.5% *n* = 85, *p* = 0.006).

**Table 1:** Demographic and clinical characteristics of patients before and after the start of PPC program (*n* = 257)

Characteristics	Oncology Alone Group (2013–2017)	Oncology and PPC Group (2018–2022)	*P* value
	n= (136)	n= (121)	
Sex Male, n (%)	84 (61.8)	67 (55.4)	0.299
Age [Years], median (IQR)	10 (4–14)	10 (5–15)	0.670
Hospital stay [days], median (IQR)	18 (7–42)	18 (7–58)	0.470
Type of cancer, n (%)			
Leukemia	65 (47.8)	54 (44.6)	0.611
Lymphoma	4 (2.9)	3 (2.5)	0.820
Solid Tumor	36 (26.5)	31 (25.6)	0.877
Central Nervous System Tumor	26 (19.1)	25 (20.7)	0.757
Rare Childhood Malignancy	5 (3.7)	8 (6.6)	0.284
Treatment goal offered for cancer, n (%)			
Palliative	57 (41.9)	50 (41.3)	0.924
Curative	79 (58.1)	71 (58.7)	0.924
Signs and symptoms 72 h before death, n (%)			
Pain	67 (49.3)	56 (46.3)	0.633
Dyspnea	73 (53.7)	66 (54.5)	0.889
Seizures	34 (25)	25 (20.7)	0.409
Agitation	30 (22.1)	13 (10.7)	0.015
Anxiety	18 (13.2)	6 (4.9)	0.023
Intervention, n (%)			
Psychology Consultation^a^	115 (84.6)	114 (94.2)	0.013
Social Work Consultation^a^	74 (54.4)	85 (70.2)	0.009
Advance Care Planning	85 (62.5)	96 (79.3)	0.006
Use of Opioids	120 (88.2)	110 (90.9)	0.485

^a^The type of psychology and social work intervention is described according to the cohort: Oncology Alone Group: psychosocial consultation model; Oncology and PPC group: psychosocial integrated model

IQR: interquartile range

Table 2 shows the mode and location of death before and after the creation of the PPC program. There were no statistically significant differences between the groups. However, some trends can be highlighted. For example, 62.8% of patients in the concurrent oncology and palliative care group received limited life support (*n* = 76). This included both withdrawal or withholding of life support and do-not-resuscitate orders. In addition, the group of patients who received care by oncology concurrent with palliative care had an increase in the number of patients who died in the general hospital ward (40.5%, *n* = 49) and a decrease in the number of patients who died in the intensive care unit (52.9, *n* = 64 vs. 58.8, *n* = 80) compared to the oncology alone group, with no statistical differences between them.

**Table 2:** Patient mode and place of death before and after starting the PPC program

	Oncology Alone group (2013–2017)	Oncology and PPC group (2018–2022)	*p* value
	(*n* = 136)	(*n* = 121)	
Mode of death, n (%)			
Life-sustaining treatment	61 (44.9)	45 (37.2)	0.213
Limited life support	75 (55.1)	76 (62.8)	0.213
Place of death, n (%)			
Inpatient hospital unit	46 (33.8)	49 (40.5)	0.269
Pediatric ICU	80 (58.8)	64 (52.9)	0.339
Emergency department	10 (7.3)	8 (6.6)	0.816

Life-sustaining treatment: cardiopulmonary resuscitation (CPR), cardioversion, intubation, or code doses of medications and life-sustaining technology. Limited life support: withdrawal or withholding of life-sustaining measures and do-not-resuscitate (DNR) orders. Withdrawal of treatment: discontinuation of the life-sustaining intervention/treatment that was already in place. Withholding treatment: ‘noninitiation’ or the decision not to escalate a life-sustaining treatment

## Discussion

To our knowledge, this is the first article that reports characteristics, patterns of care, and outcomes in children with cancer who received care prior to implementation of a PPC program in MIC versus those who received care after its implementation.

Our study shows that concurrent management in conjunction with oncology and PC favors a comprehensive approach at the EoL by addressing the biopsychosocial needs of children with cancer. The integration of PPC within pediatric cancer care has developed in response to the unique needs of children with progressive cancer and their families [23]. It refers specifically to the holistic medical, psychosocial, and spiritual care provided by an interdisciplinary team of trained professionals, with the goals of promoting QoL, mitigating suffering, supporting decision-making, assisting with care coordination, guiding end-of-life management, and addressing grief and bereavement needs [24].

The number of bothersome symptoms found in children with cancer at the end of life shows how complex and challenging care can be at that stage [25]. Agitation and anxiety were more likely addressed at the EoL when a formal PPC program was in place. This improvement may reflect the benefits of the new model of integration of pediatric palliative care with oncology care. Furthermore, high rates of ACP discussions lead most patients to receive medical care according to their values, goals, and preferences about their condition and care goals. This is in accordance with data reported by Thompkins et al., who demonstrated that (family-centered pediatric advance care planning intervention for teens with cancer) FACE-TC families significantly increased positive caregiving appraisals at 3 months postintervention [26]. Likewise, having a document specifically created for children to guide ACP can decrease anxiety and increase communication with family members [27]. In our study, we did not determine the preferences of patients or parents regarding the place of death. Further research in MICs is needed to better assess the impact of ACP on family anxiety and satisfaction with care at the end of life.

We consider it relevant to highlight that patients assessed by PPC after the implementation of the program were all concurrently followed up by a multidisciplinary team that included psychosocial interventions (nurse, psychologist, social worker, and spiritual advisor, all with specialized training in PPC and rehabilitation therapy) as well as management of physical symptoms (addressed by pediatricians and family doctors with specialized training in PPC). The importance of multidisciplinary teams (oncologists, PC, nurses, psychologists, social workers, spiritual care) when treating pediatric patients at the EoL has proven fundamentally important in preparing families to cope with the terminal phase, the moment of death, and their grief [28–30].

The lower survival of childhood cancer in MICs and LMICs is usually attributed to difficulties in treatment access due to related costs, limited presence of specialized treatment centers, delays in diagnosis, and initiation of treatment [31, 32]. In addition, these patients may suffer from other comorbidities or live in rural areas with difficult access to health facilities, which may also impact their survival [33].

It has been described that the provision of specialist PPC is associated with an increased likelihood of EoL discussions [34]. We found that the healthcare team provided ACP, allowing children and families to make decisions about their illness and striving for comfort and dignity at the EoL. Patient and family preferences were addressed in meetings during the hospital stay, and wishes were actively listened to by the palliative care team and recorded in the clinical history by the psycho-oncologist. They were socialized with the other health professionals to fulfill them. Likewise, Perez et al. described that EoL care was managed when the decision was made to limit life support, showing that in 82.8% of cases, the medical team and family participated together in the decision-making [35]. Thus, the availability of a PC unit may contribute to improvements in the quality of EoL care. Similar results were found in Peláez Cantero et al.’s study, where the existence of a palliative care team for over 5 years was more likely to be related to families voicing preferences and their fulfillment [36].

In our study, we found that nearly half of the patients in both groups received life-sustaining treatment, perhaps due to the uncertainty of imminent death, failure to adequately have an ACP, and/or the persistent intention of curative treatment, which makes it difficult to prioritize comfort measures in cancer patients in concurrent care. Nevertheless, more than half of our patients received less aggressive treatment at the EoL.

The high mortality rate in the pediatric ICU for both groups highlight the complex challenges of managing children with advanced-stage cancer. Early program implementation of PPC within oncology team may contribute to this, as both teams navigate a learning curve in providing care at this stage of the disease. This finding underscores the need for continued program development and optimization to prioritize patient center-care and family support outside the PICU [37]. Research suggest that PICU stay can negatively impact children at the end of life, potentially futile interventions (38–39), limiting family bonding [40] and incurring in unnecessary healthcare cost [41]. Interestingly, a systematic review found that two-thirds of studies on preferred place of death, reported a preference for home-based care [42]. A study in England assessed trends in place of death for children with a life-limiting condition over 14 years and found that 39,349 children died: 73% occurred in the hospital, 6% in hospice, and 16% at home [43]. It is essential to know that factors impacting decision-making for the location of care include the quality of communication and the quality of care available. Preference for the location of death in the hospital included trust in hospital staff, practical logistics, and the safety of the hospital environment [43]. This demonstrates that it is necessary to improve the EoL discussion to honor the child’s wishes and the family’s desires.

Wolfe et al. described that children with high-risk cancer experience substantial suffering throughout the course of their disease and at EoL [13]. Nevertheless, in our study, we found that the patients who received specialized PPC (Oncology and PPC group) may have benefited from integrated management and psychosocial support, and therefore had better outcomes in the control of symptoms such as anxiety and agitation, while other symptoms such as pain and dyspnea still require thoughtful consideration.

It was also noted that after the PPC program was established, increased the percentage of patients who died in the inpatient unit compared to the oncology alone group, but these results were not statistically significant. Although current evidence points to pediatric home-based PC in which the place of death should be planned [44], there is still a lack of these services in our country, perhaps because the health system does not have the availability of a home PPC team that offers a 24 h-365 day service to supply the needs at the end of life, especially when the EOL has uncontrolled symptoms, as our study demonstrates. It is necessary for health system stakeholders to define human resources and budgets for home PPC teams. Despite this, for patients who have an in-hospital death, the family accompanies the child close in an appropriate environment for a patient who is at the EoL. The PPC team in alliance with the hospital service facilitates privacy and a space to grieve. Increasing places of death outside the ICU could also lead to cost-effective and goal-aligned care regarding the location of death for children with cancer and their families [45]. The barriers that impede the transition of care to the hospital ward need to be further studied, and it remains a challenge to measure the outcomes of a specialized PPC program.

### Limitations

This is one of the first published studies with such a large population in Latin America evaluating characteristics, patterns of care, and outcomes in children with cancer before and after the implementation of a PPC program in a MIC. Nevertheless, our study has several limitations. First, it was conducted at one institution, resulting in a potential selection bias. Second, all the information is based on clinical records, and it is possible that not all the interventions for the patients were recorded accurately. Third, in the limitation of measures, we did not evaluate whether chemotherapy was discontinued in the patient. Last, although we used the total population and not a sample, the year of onset of PPC and the year 2020 relevant to the SARS-COV-2 pandemic were challenging years that could alter the results, so it would be important to explore in future follow-up studies of the palliative care program when and how would be the best approach to discontinue chemotherapy treatment in the patient at the end of life. Further studies should be implemented to verify and expand the information provided in this study.

## Conclusions

The creation of PPC programs and interventions in children with cancer is a differential marker in healthcare that promotes psychosocial support, facilitates advance care planning, and decreases symptoms of agitation and anxiety at the end of life. It is fundamental for better and more compassionate quality EoL care and patient-centered decision-making with higher symptom control and achieving goals of care. Our PPC program with the availability of a psychosocial integrative model has emerged as a paradigm of successful PC implementation and integration in a comprehensive cancer center and can serve as a model for other healthcare institutions in a middle-income country to provide quality EOL care. Further research is needed to identify appropriate metrics to demonstrate the impact of specialized PPC programs in MICs.

### Electronic supplementary material

Below is the link to the electronic supplementary material.

Supplementary Material 1

## Data Availability

All data generated or analyzed during this study are included in this published article.

## Abbreviations

ACP: Advance Care planning
CPR: Cardiopulmonary resuscitation
DNR: Do-not-resuscitate
EoL: End of life
FACE-TC: Family-centered pediatric advance care planning intervention for teens with cancer
FVL: Fundación Valle de Lili
ICU: Intensive care unit
IQR: Interquartile Range
LA: Latin America
LMICs: Low- and middle-income countries
LST: Life-sustaining treatment
MICs: Middle-income countries
NICU: Neonatal intensive care unit
PC: Palliative care
PICU: Pediatric intensive care unit
PPC: Pediatric palliative care
QoL: Quality of life
