# Supplementary material for: Children with cancer at the end of life in a middle-income country: integrated pediatric palliative care improves outcomes
Source: BMC Palliat Care. 2024 Feb 2;23:31. doi: 10.1186/s12904-024-01354-1 (PMC10836057; doi:10.1186/s12904-024-01354-1)
Supplement: Supplementary file 1 — Supplementary Material 1 [file 12904_2024_1354_MOESM1_ESM.docx]

| **Domains** | **Variables** |
| --- | --- |
| Year of age of the patients | Age in years |
| Gender | Masculine  Feminine |
| Hospital stay in days | Days of hospitalization |
| Specialty in charge of the patient in the last 72 hours of life | Oncology  Pediatric palliative care  Both |
| Type of cancer | Leukemia  Lymphoma  Solid Tumor  Central Nervous System Tumor  Rare Childhood Malignancy |
| Treatment goal offered for cancer | Curative  Palliative chemotherapy/Radiotherapy  Not specific  Supportive care |
| Place of death of the patient | Pediatric Intensive care unit  Inpatient hospital unit  Emergency department |
| Mode of death | Life-sustaining treatment  Limited life support |
| Relationship with the cause of death | Related to the disease  No related to the disease  Related to the treatment |
| Signs and symptoms 72 hours before death  Pain  Dyspnea  Seizures  Agitation  Anxiety  Others | Present or Absent |
| Does the patient receive strong opioids? | Yes  No |
| Does the patient/family receive Psychology consultation | Yes  No |
| Does the patient/family receive Social work consultation | Yes  No |
| Does the patient/family receive Advance care planning discussions | Yes  No |

Appendix 1: Care domains and variables collected for oncology alone and oncology + PPC cohort.
